# A secure online image trading system for untrusted cloud environments

**DOI:** 10.1186/s40064-015-1052-1

**Published:** 2015-06-17

**Authors:** Khairul Munadi, Fitri Arnia, Mohd Syaryadhi, Masaaki Fujiyoshi, Hitoshi Kiya

**Affiliations:** Department of Electrical Engineering, Syiah Kuala University, Jalan Tgk. Syech Abdurrauf No. 7, 23111 Banda Aceh, Indonesia; Graduate School of System Design, Tokyo Metropolitan University, 6-6 Asahigaoka, Hino-shi, Tokyo 191-0065 Japan

**Keywords:** Image trading, Image matching, Secure storage, Image scrambling, DCT, JPEG

## Abstract

In conventional image trading systems, images are usually stored unprotected on a server, rendering them vulnerable to untrusted server providers and malicious intruders. This paper proposes a conceptual image trading framework that enables secure storage and retrieval over Internet services. The process involves three parties: an image publisher, a server provider, and an image buyer. The aim is to facilitate secure storage and retrieval of original images for commercial transactions, while preventing untrusted server providers and unauthorized users from gaining access to true contents. The framework exploits the Discrete Cosine Transform (DCT) coefficients and the moment invariants of images. Original images are visually protected in the DCT domain, and stored on a repository server. Small representation of the original images, called thumbnails, are generated and made publicly accessible for browsing. When a buyer is interested in a thumbnail, he/she sends a query to retrieve the visually protected image. The thumbnails and protected images are matched using the DC component of the DCT coefficients and the moment invariant feature. After the matching process, the server returns the corresponding protected image to the buyer. However, the image remains visually protected unless a key is granted. Our target application is the online market, where publishers sell their stock images over the Internet using public cloud servers.

## Introduction

With the advancement of the Internet, multimedia content trading has become increasingly popular. As multimedia contents, such as audio, image, and video, are available in digital form, they may be benefit from ease of manipulating, duplicating, publishing, and distributing. Despite these benefits, illegal use of multimedia data tends to grow significantly unless proper protection is implemented.

One important and challenging task in multimedia content trading, including image trading, is privacy protection (Lu et al. 2009, 2010; Premaratne and Premaratne 2012; Troncoso-Pastoriza and Perez-Gonzales 2013). Most existing work in this area has focused on access control and secure data transmission (Lu et al. 2009; Iacono and Torkian 2013). The aim is to prevent unauthorized users from accessing the data and to enable secure data exchange. However, once stored on the server, the data are left unprotected. This makes the user’s private content vulnerable to untrustworthy server providers, as well as intruders.

In line with the Internet, the concept of cloud computing has also garnered increasing interest. The cloud provides computing and storage services to users via the Internet (Jeong and Park 2012). Public clouds offer these services to both organizations and individuals, but require no infrastructure or maintenance investment. Therefore, more applications and services are expected to rely on cloud resources in the future. However, privacy problems in the cloud environment need rigorous attention because the data can easily be distributed among different servers in different locations (Curran et al. 2012; Modi et al. 2013).

The Internet and cloud technology have undoubtedly pushed image trading to become commercially feasible for more individuals and small-scale business entities. Therefore, the privacy protection of image content on the cloud server is an important consideration.

Currently, various types of images—ranging from photos, to art, graphics, and historical images—are traded online in the conventional way. The trading process has been exclusively conducted over the Internet, where images can be purchased and delivered online. Nevertheless, this conventional system has a serious drawback on the server side. Images stored on the server are left unprotected, allowing illegal access and use by untrusted server providers and intruders. Hence, a new mechanism for secure online image trading is necessary.

Based on the current practices of image trading and the wide availability of cloud servers, we argue that the following requirements should be satisfied to enable a secure image trading system running in an untrusted cloud environment:

1. The system must provide privacy protection to the stored data. Images on a cloud storage should be protected such that, even if untrusted parties break the server’s access control, they cannot reach the true image content.
2. The system should provide a limited-content preview for display in various devices. To attract potential buyers, a portion of the content should be freely available for viewing. Because the display dimensions differ among devices, various reduced-size images are required.
3. The system must match the reduced-size images to the privacy-protected images.
4. The system needs to be compatible with compression standards. Because images are stored in compressed format, the image trading system should accommodate images compressed by specific standards.

Unfortunately, very few image trading schemes satisfy all these requirements. Most of the existing works (Lu et al. 2009, 2010; Premaratne and Premaratne 2012; Troncoso-Pastoriza and Perez-Gonzales 2013; Iacono and Torkian 2013; Kiya and Ito 2008; Okada et al. 2009, 2010; Liu et al. 2013; Sae-Tang et al. 2014; Zhang and Cheng 2014; Cheng et al. 2014) have separately and independently focused on a subset of these considerations.

The present paper introduces a conceptual framework for a secure image trading system in an untrusted cloud environment that satisfies all the above requirements. We focus on the Joint Photographic Experts Group (JPEG) (Wallace 1992) images, which are widely and popularly used in various applications. A trading activity involves three main parties: an image publisher, a server provider, and an image buyer. The proposed scheme facilitates secure server storage by visually protecting the publisher’s images, thus preventing access to the true image content by untrustworthy server providers and unauthorized users. Reduced-size images that serve as queries are displayed on a user interface, providing a limited-content preview for potential buyers. Our target application is the online market, in which small content publishers sell their stock images over the Internet.

The remainder of the paper is organized as follows. “Related work” briefly reviews related works in the proposed research area. “Preliminaries” introduces the preliminary information, including a review on conventional repositories for image trading and their shortcomings, the Discrete Cosine Transform (DCT) and the JPEG standard, DCT-based scrambling for visual protection, and the structural similarity (SSIM) index that measures the degree of image scrambling. “Proposed framework” describes the conceptual framework of the proposed scheme. Simulation results are presented in “Simulation results”. And, concluding remarks are given in “Conclusions”.

## Related work

The requirements formulated in “Introduction” can be divided into two main research categories: the secure storage of images on a public cloud server, and efficient image matching in visually protected (encrypted) domains for retrieval and content preview purposes.

Among the earlier works on image trading systems, the authors in Okada et al. (2009, 2010), Liu et al. (2013) proposed a framework that offers privacy or content protection. In their mechanism, an image is decomposed into two components with different levels of importance. One component is sent directly to a consumer; the other is first routed to an arbitrator or trusted third party (TTP) for fingerprinting and then sent to the consumer. This approach is impractical because of several reasons. First, the consumer receives two image components, increasing the memory and bandwidth usage. In addition, the approach requires a TTP and assumes that images are stored on a proprietary and trusted server.

An extension of the above proposal, which no longer separates an image into several components, was presented in Sae-Tang et al. (2014). This method specifically handles JPEG 2000 images. Although it removes image decomposition, it retains the TTP requirement, thus adding technical complexity to small content publishers.

Client-side encryptions for cloud storage have also been proposed (Iacono and Torkian 2013; Lu et al. 2009, 2010; Cheng et al. 2014). For instance, the approach in Iacono and Torkian (2013) encrypts the data file and changes the file structure, thus increasing the difficulties in indexing and searching of the encrypted data. In Lu et al. (2009, 2010) and Cheng et al. (2014), features are extracted from plaintext images and encrypted by the image owners. The encrypted features and images are then stored on a server equipped with a table of mapping relationship between them. When the user makes a query, the features from the plaintext query image are extracted and encrypted, and then sent to the server, where their similarity to the features encrypted in the database is calculated. This implies that feature extraction/encryption and image encryption are performed separately, incurring additional computational resources and complexities.

The histogram-based retrieval of Zhang and Cheng (2014) reduces the necessity of feature extraction/encryption. The images stored on a server are simply encrypted by permuting DCT coefficients and are compatible with the JPEG file format. Similarity between an encrypted query and an encrypted image is determined by calculating the distances of DCT coefficient histograms. However, this process requires nearly full JPEG decoding (up to inverse quantization) and proposes no mechanism for content preview. Therefore, how a potential buyer could select an image for purchase is not clarified.

An initial attempt to formulate a secure online image trading system was presented in Munadi et al. (2013), although no clear framework was described for a cloud environment context. This study also lacked a descriptive comparison with a conventional image trading system. Moreover, the experiments and analysis were based on a small dataset.

**Figure 1:**
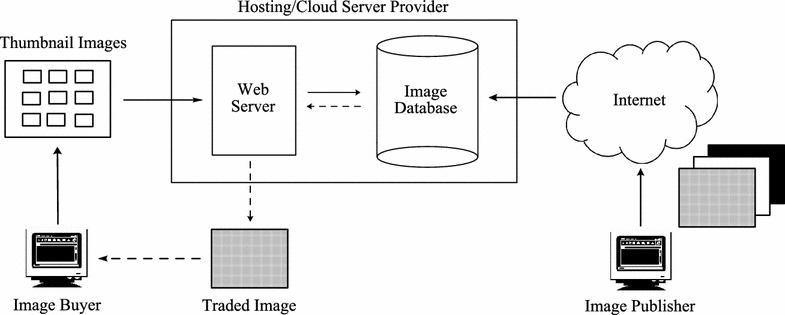
A conventional image trading system.

## Preliminaries

In this section, we present some background information that is necessary to formulate our proposed framework, including a review of conventional image trading systems and their shortcomings, the DCT and JPEG standard, image scrambling in the DCT domain, and the SSIM index, which measures the degree of scrambling.

### Conventional model of image trading

Most current applications that enable commercial transaction of images are strongly reliant on access control. Buyers obtain privileged access to the image repository after payment. Figure 1 illustrates a typical image repository and trading system in a conventional approach. An image publisher normally uses third-party services to host his/her commercial images. Potential buyers can browse a thumbnail collection, which provides small representations of the images. If the buyer is pleased with the image, he/she will pay an agreed price and receive an access key in return. The buyer will then be able to download the original size or full-resolution image. Alternatively, the image can be electronically sent to the buyer by the server. A practical application of this concept is best described by the digital image libraries available on several websites  (KITLV; Getty Images; Corbis; iStock).

**Figure 2:**
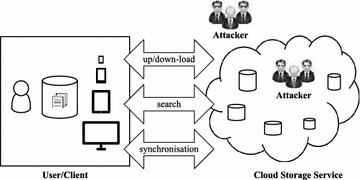
Source of threats/attacks in a cloud storage service, adapted from Iacono and Torkian (2013).

In terms of privacy, this conventional scheme is confronted with at least two serious threats or attacks that can be originated from internal and external sources, as depicted in Figure 2. The types of threats/attacks can be described as follows:

1. *External threats* Unauthorized users present an external threat to the image repository. Illegal access may be obtained under various conditions, such as lack of authentication, weak access control, and malicious attacks. When access is obtained by an unauthorized user, it becomes difficult to prevent illegal use of the images.
2. *Internal threats* A server provider often has the highest access privileges for the stored data, such as commercial images, with no risk of detection. Therefore, a malicious provider presents an internal threat to the stored data, leading to the illegal use of images, such as theft or illegal distribution.

A cloud-based image trading framework that considers the above-mentioned issues is proposed herein. It facilitates secure storage and retrieval of original images, and prevents unauthorized parties from accessing the true content of images.

### DCT and JPEG

**Figure 3:**
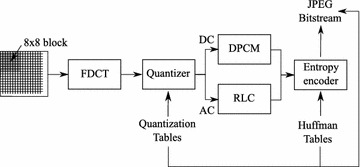
JPEG encoder.

The JPEG compression standard is based on the DCT that transforms spatial data into the frequency domain. The encoding procedure is illustrated in Figure 3 and can be summarized as follows. An original image is partitioned into 8×8 non-overlapped blocks. A function of two-dimensional Forward Discrete Cosine Transform (FDCT), as in Eq. (1), is applied to each block, resulting in 1 DC and 63 AC coefficients.

1 $$\begin{aligned} F_{uv} = \frac{C_{u}C_{v}}{4}\sum _{i=0}^{7}\sum _{j=0}^{7}\cos \frac{(2i+1)u\pi }{16}\cos \frac{(2j+1)v\pi }{16}f(i,j) \end{aligned}$$

where

$$\begin{aligned} C_{u},C_{v} = \left\{ \begin{array}{l l} \frac{1}{\sqrt{2}} &{} \quad \text {for}\,\,u,v = 0 \\ 1 &{} \quad \text {otherwise} \end{array} \right. \end{aligned}$$

For coding, an 8×8 array of the DCT coefficients is reorganized into a one-dimensional list based on a zigzag order. The order is initially started with the DC coefficient, and places the coefficients with the lowest spatial frequencies in lower indices. Note that higher-frequency components generally represent the fine details of an image, and are less sensitive to human vision. Hence, they can be quantized more coarsely than the lower frequency components, and may be discarded with negligible effect on image quality. After quantization, Differential Pulse Code Modulation (DPCM) is applied to the DC coefficient, and the AC coefficients are run-length coded (RLC). As a final stage, all the coefficients are entropy encoded using Huffman or arithmetic coding. The output of the entropy encoder and some additional information, such as header and markers, form the JPEG bitstream.

### DCT based scrambling

**Figure 4:**
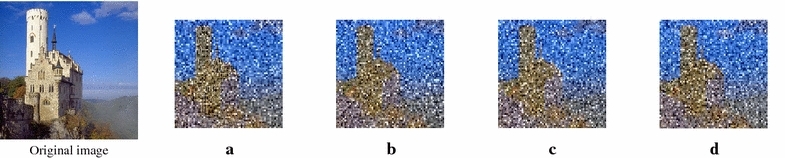
Sample of scrambled images.** a** DC coefficients are scrambled, $SSIM=0.3882.$ **b** Blocks of AC coefficient are scrambled, $SSIM = 0.1464.$ **c** Blocks of $8\times 8$ coefficients are scrambled, $SSIM = 0.1519.$ **d** DC and AC coefficients are separately scrambled, $SSIM = 0.1435.$

There are several approaches to visually protect the images, either in the spatial or transformed domain. Because we are dealing with the JPEG-coded images, it is preferable to consider available techniques that work in the DCT domain, such as those proposed in Weng and Preneel (2007), Khan et al. (2010a, b) and Torrubia and Mora (2003). These methods exploit the DCT coefficients to achieve various degrees of perceptual degradation, either by scrambling blocks of coefficients, or scrambling the individual DC and AC coefficients independently. The scrambling process can be further combined with an encryption technique to increase the level of protection.

The degree of perceptual degradation itself can be measured using the SSIM index. Assuming two images, $X$ and $Y$, as the comparison objects, the SSIM index is defined as follows (Wang et al. 2004; Weng and Preneel 2007):

2 $$\begin{aligned} SSIM(X,Y) = [l(X,Y)]^\alpha \cdot [c(X,Y)]^\beta \cdot [s(X,Y)]^\gamma \end{aligned}$$

where $X$ represents the original image and $Y$ represents the scrambled version of the original image. Functions $l()$, $c()$, and $s()$ correspond to luminance, contrast, and structural similarity, respectively, and $\alpha$, $\beta$, and $\gamma$ are the weighting factors. A simplified form of the SSIM index can be written as:

3 $$\begin{aligned} SSIM(X,Y) = \frac{(2\mu _{X}\mu _{Y}+C_{1})(2\sigma _{XY}+C_{2})}{(\mu _{X}^2+\mu _{Y}^2+C_{1})(\sigma _{X}^2+\sigma _{Y}^2+C_{2})} \end{aligned}$$

where $\mu$ is the mean intensity, $\sigma$ represents the (co)variance, and $C_{1}$, $C_{2}$ are numerical stability constants (Wang et al. 2004; Weng and Preneel 2007). The value of SSIM ranges from 0 to 1, with a value of 1 indicating that $X$ and $Y$ are identical.

Samples of DCT-based scrambled images with their respective SSIM values are shown in Figure 4. As shown, different degrees of visual degradation can be obtained by applying different arrangements of the DCT coefficients. The image with the lowest SSIM value is considered the most visually protected.

## Proposed framework

**Figure 5:**
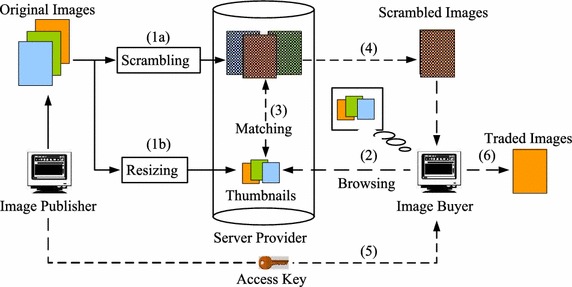
Proposed framework.

In this section, we describe a conceptual image trading framework for an untrusted cloud environment that satisfies all the requirements mentioned in “Introduction”. The proposed framework enables secure online trading, and allows the images to be securely stored on the cloud servers after being visually protected and to be retrieved in their protected state. The following description is based on the scheme illustrated in Figure 5.

Original images owned by an image publisher are first encoded and visually protected by means of scrambling in the DCT domain (1a). At the same time, thumbnails are generated by resizing the original images to any required sizes for viewing in a display device (1b). The protected images are then uploaded and stored on a cloud repository server. In this manner, the true visual content of the original images cannot be accessed by the server provider. Thumbnails can be stored on the same server, and are publicly accessible through the website. A potential image buyer will browse the thumbnail library and choose images of interest, which also serve as queries (2). When a query image is submitted, the thumbnail is matched with the protected images by comparing the moment invariants of the thumbnail and of the DC-image generated from the protected images (3). After this matching process, the server will return the matched image, which can then be downloaded or sent to the potential buyer (4). However, the matched image remains visually protected unless a key is granted by the image publisher after payment or other authorization (5). Using an authentic key, the buyer will be able to decode and descramble the data, resulting in the true traded image (6).

### Scrambling process

**Figure 6:**
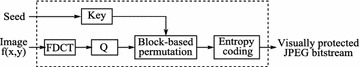
A simplified diagram of JPEG-based image scrambling.

The main purpose of image scrambling is to provide visual protection so that the true content is perceptually meaningless or degraded. Therefore, the images are secure against ill-intentioned parties who may have access to the server, such as a hosting provider or hackers. Depending on the degree of scrambling, visual protection can be achieved by applying existing scrambling techniques that work in the DCT domain, such as those proposed in Kiya and Ito (2008) and Khan et al. (2010a, b).

A simplified diagram of a JPEG-based image scrambling for visual protection is shown in Figure 6, in which a block-based permutation is applied to the quantized DCT coefficients. Descrambling is simply a reverse process, given the same key as in the scrambling proses is available.

### DC image generation and thumbnails

**Figure 7:**
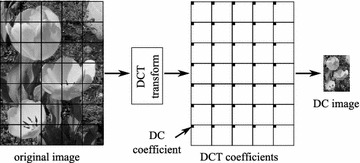
DC-image generation.

It is known that the DC coefficient of each 8×8 array of DCT coefficients is actually an average value of the 64 pixels within the corresponding block. Hence, it contains very rich visual information. An image constructed from DC components is a reduced-sized version that is visually similar to the original. Therefore, the DC image itself is a rich feature descriptor that can be exploited for matching purposes.

The process of generating a DC-image from DCT coefficients is illustrated in Figure 7. Initially, an image is partitioned into 8×8 non-overlapped blocks (referred to as a tile or a block), and a forward DCT function is employed to each block. The DC coefficient of each block represents the local average intensity and holds most of the block energy. DC coefficients from all of the blocks are then arranged according to the order of the original blocks, resulting in a reduced-size image ($\frac{1}{64}$ of the original image) referred to as a DC-image.

In relation to the JPEG standard, it is worth noting that the DC coefficients can be directly extracted from the JPEG bitstream without the need for full JPEG decoding (Arnia et al. 2009), and the DC-image can be generated accordingly.

However, thumbnails for preview or browsing purposes can be produced by downscaling the original images to the sizes best suited to the dimensions of the display devices.

### Image matching

In this section, an image matching technique and its corresponding matching distance are described. We exploit the seven Hu moments (Ming-Kuei 1962) for matching purposes. The moments of an image, with pixel intensities $I(x,y)$ and of size $M\times N$, are defined by:

4 $$\begin{aligned} m_{pq} = \sum _{y=0}^{M-1} \sum _{x=0}^{N-1}x^py^qI(x,y) \end{aligned}$$

Rather than Eq. (4), the central moments:

5 $$\begin{aligned} \mu _{pq} = \sum _{y=0}^{M-1} \sum _{x=0}^{N-1}(x-\bar{x})^p(y-\bar{y})^qI(x,y) \end{aligned}$$

with

$$\begin{aligned} \bar{x}=\frac{m_{10}}{m_{00}}, \quad \bar{y}=\frac{m_{01}}{m_{00}} \end{aligned}$$

are often used, which are invariant to translation. Furthermore, normalized central moments are defined by:

6 $$\begin{aligned} \eta _{pq} = \frac{\mu _{pq}}{\mu ^\gamma _{00}} \end{aligned}$$

with

$$\begin{aligned} \gamma = \frac{p+q}{2} + 1,\quad p+q=2,3,\dots \end{aligned}$$

and these are also invariant to changes in scale. Algebraic combinations of these moments can provide more attractive features. The most popular are those offered by Hu, which are independent of various transformations. Hu’s original moment invariants (Ming-Kuei 1962; Huang and Leng 2010) are given by:

$$\begin{aligned}M_{1} &= \mu _{20} + \mu _{02}\\M_{2} &= \left( \mu _{20} - \mu _{02}\right) ^2 + 4\mu ^2_{11}\\M_{3} &= \left( \mu _{30} - 3\mu _{12}\right) ^2 + 3\left( \mu _{21} + \mu _{03}\right) ^2\\M_{4} &= (\mu _{30} + \mu _{12})^2 + (\mu _{21} + \mu _{03})^2\\M_{5} &= (\mu _{30} - 3\mu _{12})(\mu _{30} + \mu _{12})[(\mu _{30} \\&\quad + \mu _{12})^2 - 3(\mu _{21} + \mu _{03})^2] \\&\quad + 3(\mu _{21} - \mu _{03}[3(\mu _{30} + \mu _{12})^2 - (\mu _{21} + \mu _{03})^2] \\M_{6} &= (\mu _{20} - \mu _{02})[(\mu _{30} + \mu _{12})^2 \\&\quad - (\mu _{21} + \mu _{03})^2] + 4\mu _{11}(\mu _{30} \\&\quad + \mu _{12})(\mu _{21} + \mu _{03}) \\M_{7} &= (3\mu _{21} - \mu _{03})(\mu _{30} + \mu _{12}) \\&\quad\times \left[ (\mu _{30} + \mu _{12})^2 - 3(\mu _{21} + \mu _{03})^2\right] \\&\quad+(\mu _{30} - 3\mu _{12})(\mu _{21} + \mu _{03})[3(\mu _{30} + \mu _{12})^2 - (\mu _{21} + \mu _{03})^2] \end{aligned}$$

Image matching, between thumbnails and visually protected images, involves calculating the moment distance, $d$, between the thumbnails and the DC component of the visually protected images. We define the distance as:

7 $$\begin{aligned} d(a,b) = \sum _{j=1}^{7}|M_j^a - M_j^b| \end{aligned}$$

where, $a$ and $b$ denote the thumbnail and the DC image, respectively, and $M$ represents Hu’s moments. The matching process proceeds as follows:

1. The moments of a thumbnail image are calculated.
2. DC coefficients from each block of the visually protected JPEG bitstream are extracted to generate the DC image.
3. The moments of the DC images are calculated.
4. The moment distances between the query and the DC images are calculated using Eq. (7). The minimum value of $d(a,b)$ corresponds to image matching.

### Key sharing

Once authorization has been requested, a corresponding scramble key is sent to the buyer by the image publisher. The true image content is accessible to the image buyer after proper decoding that includes the unscrambling process using the given key. Various options are available for delivering the scramble key to a buyer. For instance, it could be attached to the system and use the same cloud server or a system built in a different and independent server, or could be accomplished by other online means, such as email.

**Figure 8:**
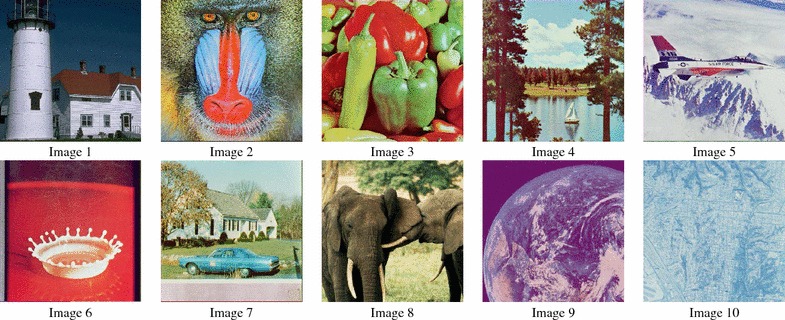
Ten sample images taken from a dataset of 100 images and used as queries in the simulation.

## Simulation results

Simulations were mainly conducted to verify the matching performance between thumbnails of various sizes that serve as query images and their corresponding DC-images extracted from the visually protected images. These images were assumed to be stored on the server and available for trading. The moment distance defined in Eq. (7) was used as the matching metric.

### Simulation conditions

The experiment was conducted using a dataset of 100 images with an original size of 512 × 512 pixels. Ten samples used as query images are shown in Figure 8. Using four different thumbnail sizes for viewing, four separate experiments were carried out. In each experiment, thumbnails were generated by rescaling the original images by a factor of 0.125, 0.1875, 0.25, and 0.391. This resulted in images of size 64 × 64, 96 × 96, 128 × 128, and 200 × 200 pixels, respectively.

As described in “DCT based scrambling”, block-based scrambling of the DCT coefficients was performed to produce visually protected images. For simplicity, we scrambled only blocks of AC coefficients while preserving the original position of the DC coefficients. The size of the DC images, constructed using the DC coefficients of the protected images, was 64 × 64 pixels. These protected images and thumbnails were assumed to be stored on the same server.

**Figure 9:**
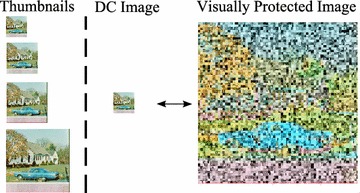
Scaled size of thumbnails with different dimensions, a DC-image, and a visually protected image.

Figure 9 shows an example of the images generated in the simulations. The image size was scaled to represent thumbnails for content preview (browsing), a DC image, and a visually protected image. For comparison purposes, we also calculated the distance between the thumbnails and the visually protected images.

### Results

**Table 1:** Distance values of the matching process between 10 thumbnails (query images) and their corresponding visually protected images. The thumbnail size was 64 × 64 pixels

		Thumbnails (queries)									
		Image 1	Image 2	Image 3	Image 4	Image 5	Image 6	Image 7	Image 8	Image 9	Image 10
**Visually protected images**											
**Image 1**		39.7189	13.7239	32.6606	39.1072	16.0546	38.3149	26.1712	24.3939	32.9669	13.0074
**Image 2**		74.6377	39.1847	45.9280	42.6521	51.1529	47.1137	42.1569	11.2891	55.4061	29.9876
**Image 3**		46.0236	21.9212	25.3841	45.8017	35.9153	30.9343	21.1973	5.6480	51.804	3.9774
**Image 4**		51.8784	23.7847	47.4008	56.1663	28.6371	46.6445	14.9005	6.4785	50.0290	12.3105
**Image 5**		81.8966	34.1338	77.5687	54.1198	55.4902	68.1009	43.1274	16.1991	52.1721	26.5318
**Image 6**		46.7303	22.7267	36.2143	49.1851	17.6216	46.8785	29.0792	9.9819	36.8837	5.2726
**Image 7**		72.3930	45.8260	65.1096	52.7709	48.9887	60.1583	36.2682	12.3805	71.6876	7.0817
**Image 8**		98.7694	49.9285	84.5833	76.0566	58.5684	85.4906	47.4281	45.9970	79.7815	31.1651
**Image 9**		59.3159	23.1605	35.8060	50.8272	32.1720	51.2894	26.2252	6.8737	46.0803	5.6870
**Image 10**		77.8870	62.6029	66.7996	71.1085	71.4843	88.5928	59.5243	49.7886	73.1289	36.0710

**Table 2:** Distance values of the matching process between 10 thumbnails (query images) and their corresponding visually protected images. The thumbnail size was 200 × 200 pixels

		Thumbnails (queries)									
		Image 1	Image 2	Image 3	Image 4	Image 5	Image 6	Image 7	Image 8	Image 9	Image 10
**Visually protected images**											
**Image 1**		61.5043	17.1736	35.4991	58.3845	24.3850	34.1974	16.8766	9.6885	40.4713	6.1305
**Image 2**		73.7492	28.0326	59.4003	47.3329	43.9759	87.7005	40.2979	13.3583	48.8846	8.0870
**Image 3**		52.0151	27.3603	37.5624	43.0131	43.3199	54.9353	27.2397	8.5423	38.8602	3.1863
**Image 4**		50.3825	28.9227	37.8932	44.1265	36.5985	51.9405	24.1728	6.8419	43.6390	13.5317
**Image 5**		73.5381	38.7909	48.0761	60.4244	51.4285	73.3088	35.5317	15.2689	59.8085	19.8409
**Image 6**		43.2402	32.3024	30.7378	43.4939	18.9351	40.6727	16.9871	14.1854	37.9260	9.3787
**Image 7**		70.5254	37.1785	48.5439	45.5184	36.7016	60.7536	36.9330	19.5183	58.4514	18.9622
**Image 8**		83.9371	58.4279	71.4194	83.1694	59.6877	79.7380	51.4882	31.0528	71.7856	46.3062
**Image 9**		47.7183	24.7508	47.6969	36.3379	26.7633	45.0160	15.7848	6.5863	53.1393	7.2226
**Image 10**		107.0481	57.1520	82.9739	81.9254	74.0595	89.6267	52.5808	41.8910	85.5333	42.6746

**Table 3:** Distance values of the matching process between 10 thumbnails (query images) and their corresponding DC-images. The thumbnail size was 64 × 64 pixels

		Thumbnails (queries)									
		Image 1	Image 2	Image 3	Image 4	Image 5	Image 6	Image 7	Image 8	Image 9	Image 10
**DC images**											
**Image 1**		**0.0308**	26.1828	12.3192	9.5167	21.0654	6.7512	28.1004	44.6903	9.1557	45.9784
**Image 2**		26.1424	**0.0457**	13.8484	19.9118	10.9063	23.1223	5.0284	18.5409	17.5827	19.8290
**Image 3**		12.2702	13.9234	**0.0435**	8.9586	8.9877	14.1178	15.8231	32.4131	4.6868	33.7012
**Image 4**		9.5371	19.8877	8.9650	**0.0299**	12.5154	10.2579	19.3329	35.1462	7.2646	36.4344
**Image 5**		21.0848	10.8786	8.9820	12.5459	**0.0272**	19.6494	7.6424	23.5985	12.5251	24.9530
**Image 6**		6.7220	23.1509	14.1523	10.2408	19.6186	**0.0223**	25.0684	41.6584	12.2429	42.9465
**Image 7**		28.2207	5.1106	15.9084	19.5040	7.6054	25.2006	**0.1466**	16.4626	19.6610	17.7508
**Image 8**		44.8671	18.6913	32.5548	35.3574	23.8087	41.8470	16.7737	**0.1949**	36.3074	5.6874
**Image 9**		9.1404	17.6111	4.6870	7.2635	12.4937	12.2450	19.5287	36.1186	**0.0249**	37.4068
**Image 10**		45.9559	19.7801	33.6437	36.4462	24.9648	42.9359	17.8626	5.6818	37.3962	**0.0155**

**Table 4:** Distance values of the matching process between 10 thumbnails (query images) and their corresponding DC-images. The thumbnail size was 200 × 200 pixels

		Thumbnails (queries)									
		Image 1	Image 2	Image 3	Image 4	Image 5	Image 6	Image 7	Image 8	Image 9	Image 10
**DC images**											
**Image 1**		**0.0395**	26.1341	12.2620	9.5288	21.0766	6.7601	28.2124	44.8588	9.1255	45.9477
**Image 2**		26.0971	**0.0676**	13.8381	19.8279	10.8827	23.0651	5.1133	18.7770	17.5254	19.8659
**Image 3**		12.2658	13.9006	**0.0262**	8.9295	9.0135	14.1018	15.9619	32.6083	4.6485	33.6972
**Image 4**		9.4581	19.8711	8.8703	**0.0969**	12.5077	10.1546	19.4658	35.4160	7.2475	36.5049
**Image 5**		21.1033	10.9176	9.0168	12.4646	**0.0592**	19.6605	7.5280	23.7708	12.5316	24.9239
**Image 6**		6.7704	23.0348	14.0701	10.2606	19.6018	**0.0833**	25.1131	41.7595	12.1988	42.8484
**Image 7**		28.2869	5.1680	16.0096	19.5581	7.4639	25.2549	**0.1397**	16.5872	19.7152	17.6761
**Image 8**		45.0437	18.8943	32.7664	35.4996	23.9518	42.0117	16.8160	**0.1736**	36.4720	5.6394
**Image 9**		9.1992	17.5245	4.6990	7.2693	12.4669	12.2871	19.6027	36.2492	**0.0723**	37.3380
**Image 10**		45.9351	19.7857	33.6578	36.3910	24.9098	42.9031	17.7074	5.6642	37.3634	**0.0394**

The results of each set of query images are presented in Tables 1, 2, 3 and 4. There are 100 matching runs presented in each table. The first two tables present the matching distances between the thumbnails (query images) and the visually protected images, and the last two present the matching distances between the thumbnails (query images) and the DC images generated from the visually protected images. Simulations using a dataset of 100 images with four different sizes of query images resulted in 40,000 matching attempts between the thumbnails and the visually protected images, and 40,000 matching attempts between the thumbnails and the DC images.

In Tables 1 and 2, we present the matching distances between the thumbnails and visually protected images. The sizes of the thumbnails are 64 × 64 and 200 × 200 pixels, respectively. As can be seen, the distance values vary and are much higher than zero. These results confirmed that the visual content of the thumbnails and of their corresponding visually protected images is no longer identical after DCT-based scrambling. Moreover, the proposed distance measure is not applicable to a direct matching between a thumbnail and a visually protected image.

Table 3 summarizes the matching results between the thumbnail and the DC images of the same size. In this case, the displayed image for browsing and the DC image generated from the visually protected image were the same size, i.e., 64 × 64 pixels. In contrast to the above results, the distances between the thumbnails and their corresponding DC images were very close to zero (bold values), i.e., less than 0.2.

The matching results between the thumbnail and DC images of different sizes are presented in Table 4. In this case, the thumbnail took its largest size, 200 × 200 pixels, whereas the size of the DC image was 64 × 64 pixels. Similar to the results in Table 3, the distance values were very small (bold values). Note that the distance values between all thumbnails of various sizes and the DC images were close to zero. This is confirmed by the averaged value of all the matching distances, as presented in Table 5.

From the above results, we can make several concluding observations. Despite its simplicity, the proposed system offers both visual protection and a content preview of the traded images. The proposed moment distance performed satisfactorily in retrieving the target images, with all queries for each experiment returning the correct visually protected images. This means that the matching performance was not affected by the variation in thumbnail size. Thus, thumbnails could be adjusted according to the size of display device.

**Table 5:** Averaged distance values between all the thumbnails (query images) of various sizes and their corresponding DC images

Thumbnail size			
$64\times 64$	$96\times 96$	$128\times 128$	$200\times 200$
0.0548	0.0449	0.0648	0.0635

Each size represents an average of 100 values.

## Conclusions

We have presented a conceptual framework for secure online image trading in a cloud environment. The traded images were visually protected in the DCT domain, and stored on an untrusted server. Thumbnails of original images were publicly accessible through the website and served as queries. Image matching between the thumbnails and protected images was achieved by comparing the moment invariants of the thumbnails and of the DC-image generated from the protected images. The proposed moment distance enabled the target images to be differentiated from other protected images in the database.
